# Relationships between Toenail, Urinary, and Drinking-water Fluoride Concentrations in a Pregnancy Cohort using Private Water Systems in the United States

**DOI:** 10.1007/s12011-026-05182-7

**Published:** 2026-06-17

**Authors:** Guillermo Tamayo-Cabeza, Esperanza Angeles Martínez-Mier, Gina A. Castiblanco-Rubio, Frank Lippert, Janet L. Peacock, Christine Till, Carly V. Goodman, David B. Flora, Bruce P. Lanphear, Susan A. Korrick, Margaret R. Karagas

**Affiliations:** 1 Department of Dental Public Health and Dental Informatics, School of Dentistry, Indiana University Indianapolis, Indianapolis, IN USA; 2 Department of Sociology, College of Liberal Arts, Purdue University, Indianapolis, IN USA; 3 Department of Biomedical and Applied Sciences, Oral Health Research Institute, School of Dentistry, Indiana University Indianapolis, Indianapolis, IN USA; 4 Department of Epidemiology, Geisel School of Medicine, Dartmouth College, Hanover, NH, USA; 5 Department of Psychology, York University, Toronto, ON Canada; 6 Faculty of Health Sciences, Simon Fraser University, Burnaby, BC Canada; 7 Channing Division of Network Medicine, Department of Medicine, Brigham and Women’s Hospital, Boston, MA USA

**Keywords:** Fluoride, Water, Toenail, Urine, Pregnancy, Biomarker

## Abstract

To assess the strength of the relationships between fluoride concentrations in tap water, toenails, and urine in a U.S. cohort of pregnant participants using private, unregulated water systems. We analyzed fluoride concentrations in residential tap water directly with a fluoride-specific electrode, and in toenail and spot urine samples using a micro-diffusion technique. We collected samples from 436 pregnant women in the New Hampshire Birth Cohort Study. We used Spearman’s rank correlation to assess relationships between fluoride concentrations in water, urine and toenails, stratified by self-reported percent of residential tap water use and residential water filtration practices. In sensitivity analyses, we re-assessed these relationships by excluding participants who reported tea consumption during pregnancy. Median (IQR) fluoride concentrations were 0.19 (0.38) mg/L for tap water, 2.60 (1.71) µg/g for toenails, and 0.39 (0.38) mg/L for specific gravity-adjusted urine. For 18% of participants, tap water fluoride concentrations exceeded the U.S. Public Health Service recommended 0.7 mg/L. Fluoride concentration in tap water was correlated with that of toenails (rho = 0.31, *p* < 0.01) and urine (rho = 0.18, *p* < 0.01). These correlations were stronger for participants reporting higher household tap water use and no residential filtration. After excluding tea drinkers, the water-urinary fluoride correlation increased more markedly than the correlation between water and toenail fluoride. Fluoride in tap water was positively correlated with urinary and toenail fluoride levels among pregnant participants, with the overall strongest correlation observed for toenails. Our findings support the potential of toenail fluoride as a reliable long-term biomarker for assessing fluoride exposure during pregnancy.

**Supplementary Information:**

The online version contains supplementary material available at 10.1007/s12011-026-05182-7.

## Introduction

Fluoride is a naturally occurring mineral that is widely recognized for its role in preventing dental caries [1]. In the United States, community water fluoridation (CWF) is a common practice, with nearly three-fourths of the population on public water systems receiving water adjusted to a federally recommended fluoride concentration [2]. While fluoride is beneficial for preventing dental caries, concerns over increasing systemic fluoride exposure and rising rates of enamel fluorosis led the U.S. Public Health Service to advocate for lowering the recommended fluoride concentration in drinking water from 0.7 to 1.2 mg/L to 0.7 mg/L in 2015 [3]. However, in some geographic areas [4], groundwater wells or private unregulated wells may contain high levels of naturally occurring fluoride, posing a risk of excessive exposure [5]. The risk of excessive exposure is of particular concern during pregnancy, as fluoride readily crosses the placenta and reaches the fetal brain [6]. A growing body of research suggests fluoride is a developmental neurotoxicant [7, 8]. For instance, studies have associated higher maternal fluoride exposure during pregnancy with adverse neurodevelopmental outcomes in offspring, including lower intelligence quotient (IQ) scores [9–12] and increased symptoms of attention-deficit/hyperactivity disorder (ADHD) [13, 14]. Given the potential for fetal neurotoxicity, establishing valid and reliable biomarkers of prenatal exposure is essential [15]. Maternal urinary fluoride, a measure of recent fluoride exposure [16], is the most commonly used biomarker in prior studies of developmental neurotoxicity of fluoride [8]. Weak to moderate positive correlations have been reported between maternal spot urinary fluoride concentrations (adjusted for dilution) and community water fluoride levels [17, 18]. In a Canadian study of 1,566 pregnant women, the correlation between water fluoride concentration and urinary fluoride averaged across three trimesters was 0.49 [17]. Similarly, a study of 965 pregnant women residing in Kansas, Missouri, Ohio, Kentucky, and Indiana [19] reported a correlation of 0.30 between urinary fluoride measured in a single spot sample and water fluoride levels, while a smaller California study of 47 pregnant women found a correlation of 0.21 [18]. Despite these consistent positive correlations, spot urine samples have important limitations, as fluoride excretion can fluctuate in response to recent intake, hydration status, dietary influences [20], and pregnancy-related factors [6], thereby increasing within person variability in exposure estimation.

Toenails are a more appropriate biomarker for estimating longer-term fluoride exposure compared to urine or plasma [21]. Toenails are preferred over fingernails due to their slower growth rate and lower potential for external contamination [21]. Fluoride is incorporated into toenails during their growth, reflecting cumulative fluoride systemic concentrations over a protracted period of approximately 3 to 4 months [22, 23]. Based on this exposure window, maternal toenail samples collected at 24–28 weeks’ gestation (approximately the second trimester) and at 6 weeks postpartum can provide cumulative measures of fluoride exposure during the early- and late-prenatal periods, respectively.

Previous studies have demonstrated a positive association between fluoride content in toenails and fluoride in water, especially in communities with higher levels (e.g. exceeding 1.5 mg/L) [24, 25]. However, no studies to our knowledge have examined toenail fluoride from pregnant women and compared them directly with urinary and water fluoride levels, especially in communities with a high degree of variation of naturally occurring fluoride in water [26].

While drinking water is often considered the primary pathway for fluoride exposure in humans [27], contributions to total fluoride exposure can also arise from the ingestion of dental hygiene products and high-fluoride foods and beverages, such as tea and seafood [28, 29]. Nevertheless, given that drinking water remains a primary fluoride source in communities with fluoridation [27], assessing the relationship between water fluoride concentration with that in toenail and urinary samples can contribute to their reliability as fluoride exposure biomarkers in this population. The objective of this study was to assess the strength of the relationships between fluoride concentrations in tap water, toenails, and urine in a U.S. cohort of pregnant participants using private, unregulated water systems. Analyses were further stratified by the percent that participants used their residential tap water and by their residential water filtration practices.

## Methods

### Study Design and Setting

We included participants in the New Hampshire Birth Cohort Study (NHBCS), an ongoing, prospective study of pregnant individuals and their children residing in rural New Hampshire, USA. The NHBCS was initially designed to investigate the effects of various environmental exposures, including elevated levels of arsenic and other contaminants in private water systems, on fetal growth and child development [30]. Recruitment began in 2009 through prenatal clinics, with participants initially enrolled between approximately 24 and 28 weeks of gestation. Original eligibility criteria for inclusion in the cohort were: (1) between 18 and 45 years of age; (2) English literacy; (3) a singleton pregnancy; (4) use of a private, unregulated water system as the primary source at their residence; (5) stable residence since their last menstrual period with the intention to remain there throughout the pregnancy.

### Participants

**Fig. 1:**
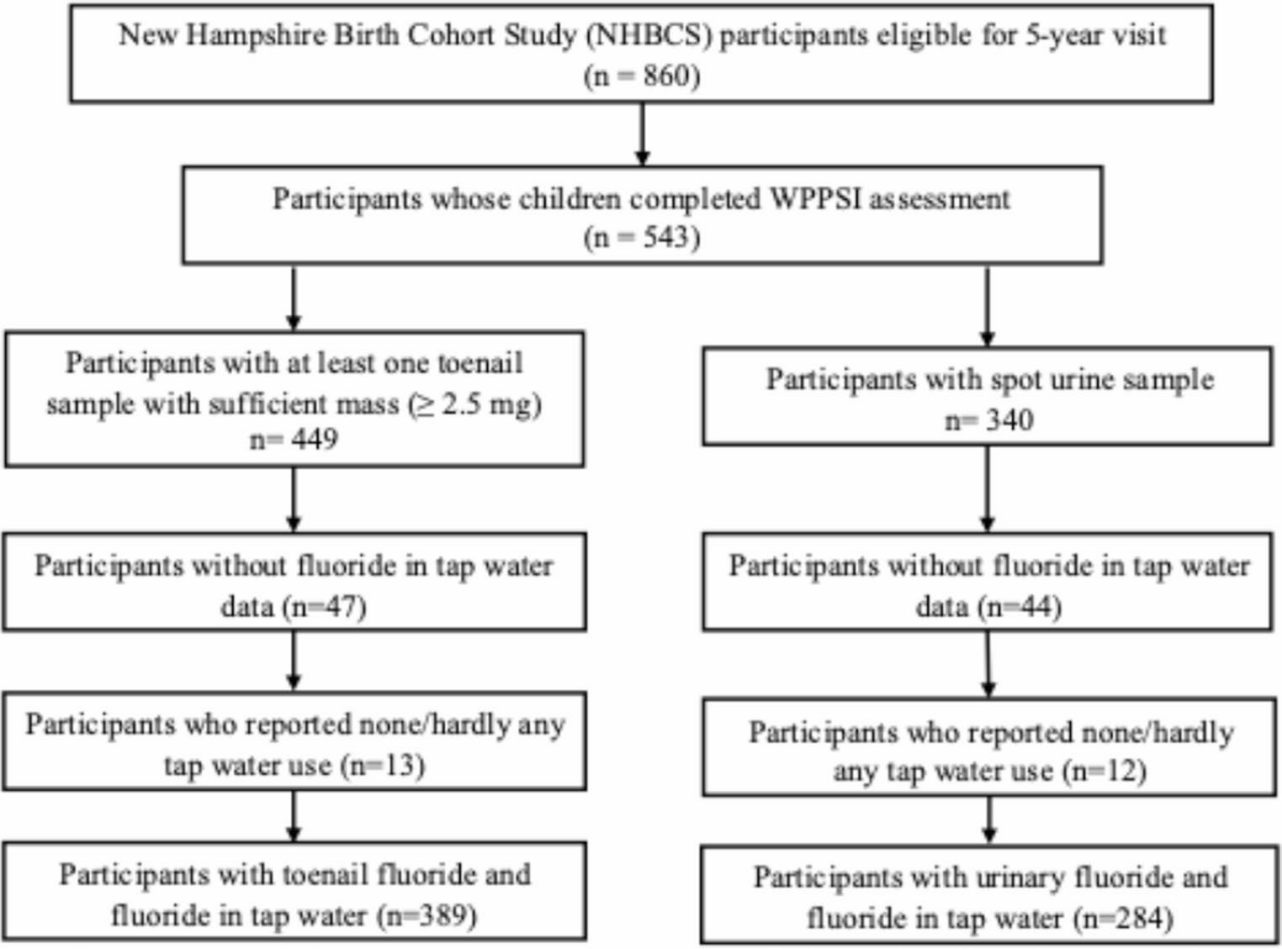
Flow diagram of participant inclusion based on toenail and urine fluoride samples availability

This study included data from a subset of the NHBCS participating in a follow-up investigation on the impact of fluoride exposure on neurodevelopment, the protocol for which is detailed elsewhere [31]. Study visits and sample collections were conducted through a combination of prenatal clinic visits and at-home collection. From the 860 participants eligible for the 5-year study visit, 543 mother-child pairs completed the Wechsler Preschool and Primary Scale of Intelligence (WPPSI) assessment. From these 543 mother-child pairs, we included mothers with available biological samples and tap water data (Fig. 1). At enrollment at approximately 24–28 weeks’ gestation (second trimester), a toenail sample (*n* = 430), a spot urine sample (*n* = 340), and a residential tap water sample (*n* = 611) were collected. At approximately 6 weeks postpartum, a second toenail sample was collected (*n* = 459). For the purpose of the current study, we used toenail clippings from the lesser toes (i.e., all toes excluding the big toe) based on the sample availability in this cohort. We included participants whose samples weighed at least 2.5 mg, consistent with a previous analysis of the reliability of fluoride measurement in toenail samples [32]. Of the enrolled cohort, participants with at least one prenatal or postpartum toenail sample were included, and an average toenail fluoride content was calculated for participants with both samples (*n* = 194), yielding a sample size of 449 (Fig. 1). Because we were interested in assessing the relationship between biomarkers and tap water fluoride concentration, we excluded those who reported none/hardly any use of household tap water during pregnancy, resulting in a final sample of 389 with toenail fluoride (only *n* = 171 participants had both prenatal and postpartum samples), 284 with urinary fluoride, and 436 with at least one water sample and any biological sample. Participants provided written informed consent, and the study was approved in accordance with the guidelines from the Committee for the Protection of Human Subjects at Dartmouth College.

### Fluoride Measurements

#### Water Fluoride

Information on tap water use during pregnancy was collected through questionnaires and was reported in a previous publication from our group [33]. Specifically, participants were asked: “Overall, what percentage of the water you use for drinking and cooking comes from your household tap water? Responses were: none/hardly any, < 25%, 25–50%, 50–75% and 75%−100%. Participants were provided with instructions and materials to collect a water sample from their tap in high density polypropylene bottles [30]. If water filters were used, participants were asked to label the sample accordingly as unfiltered or filtered sample. Forty-seven participants out of 436 (11%) provided both unfiltered and filtered water samples. A comparison between these paired filtered and unfiltered water samples showed marginally lower levels with filtration (0.15 versus 0.20 mg/L) but the difference was not statistically significant (Table S1). For these participants, only the filtered water samples were used for analyses in the present study based on the assumption that their reported residential filtration was indicative of their use. Water samples were shipped to Indiana University School of Dentistry’s fluoride laboratory for analysis. One mL of each water sample was dispensed into an appropriately labeled vial. One mL of Total Ionic Strength Adjustment Buffer (TISAB II) was added to each sample vial and then vortexed to ensure thorough mixing. Each sample was analyzed for fluoride using a fluoride ion-specific electrode (limit of detection (LOD) = 0.02 mg/L) and an ion analyzer as described previously [34]. From 436 water samples analyzed, 99.7% were above the LOD. A standard calibration was similarly prepared and used for determination of the fluoride concentration of each of the samples. Duplicate analyses on a subset of 96 samples were performed for quality control, showing high reliability (r = 0.97).

#### Toenail and Urinary Fluoride

Fluoride in toenails and urine samples were analyzed using a modification of the hexamethyldisiloxane (HMDS)-facilitated micro-diffusion technique as described by Martínez-Mier et al. [34]. Toenail samples were cleaned by sonicating in deionized water for 2 min and then dried at 37 °C for 4 h. Toenail samples’ mass used for fluoride analysis ranged from 2.5 to 19.8 mg (median = 4.8 mg; 25th percentile = 3.7 mg; 75th percentile = 6.6 mg). For urine samples, 1.0 mL aliquots of each participant’s sample were used. Each toenail or urine sample was placed at the base of a 60 × 15 mm disposable Petri dish along with 2.0 mL of deionized water (diH₂O). The interior of the corresponding lid was coated with petroleum jelly, and a trapping solution of 50 µL of 0.05 N sodium hydroxide (NaOH) was pipetted onto it in five droplets. After the dish was tightly sealed, a small hole was created in the lid with a soldering iron, and 1.0 mL of HMDS-saturated 3 N H₂SO₄ was introduced before the hole was immediately sealed with petroleum jelly. The sealed dishes were left overnight at ambient temperature to allow for the diffusion and trapping of liberated fluoride. Following this diffusion period, the NaOH trap was recovered, buffered to pH 5.2 with 25 µL of 0.1 M acetic acid, and adjusted to a final volume of 100 µL with diH₂O. Fluoride concentrations were determined using a fluoride ion-specific electrode (LOD = 0.02 mg/L for urine samples and 0.02 µg/g for toenail samples) and a pH/ISE meter, with quantification referenced against a standard curve prepared under identical conditions. All urine samples and toenail samples included in the present study were above the LOD.

#### Correction for Variations in Urine Dilution

Urine specific gravity (SG) was determined using an ATAGO^®^ Pen Refractometer that was zero-calibrated under darkened room conditions, concurrent with the setup for fluoride analysis. Urinary fluoride adjusted by specific gravity was calculated using a standard equation:

$$\:{\mathrm{UF}}_{\mathrm{SG}}\text{}\mathrm{=}\text{}{\mathrm{UF}}_{\mathrm{i}}\text{}\mathrm{*}\text{}\mathrm{(}{\mathrm{SG}}_{\mathrm{M}}\mathrm{-1)/(}{\mathrm{SG}}_{\mathrm{i}}\mathrm{-1)}$$

where UF_SG_ is the urinary fluoride adjusted by specific gravity (mg/L), UF_i_ is the observed fluoride concentration, SG_i_ is the specific gravity of the individual urine sample, and SG_M_ is the study-specific gravity median (the median specific gravity for all available urine samples) [17, 20].

### Tea Consumption

Tea is an important source of fluoride [35, 36] due to its high absorption from soil and storage in the leaves [37]. Using a validated food frequency questionnaire (FFQ) [38] that was administered at enrollment at approximately 24–28 weeks’ gestation (second trimester), we obtained information on self-reported tea consumption to perform sensitivity analyses on the relationship between fluoride concentrations in tap water, toenails and urine. The FFQ item “*Tea with caffeine (8 oz. cup)*,* including green tea*” was used to categorized participants’ tea consumption as “No” if they reported “Never, or less than once per month” consumption, and “Yes” if they reported consumption during pregnancy (≥ “1–3 per month”).

### Statistical Analysis

Descriptive statistics were used to summarize categorical variables (using frequencies and percentages) and continuous variables (using median, interquartile range [IQR], and percentiles). The distribution of fluoride in tap water, toenail and urine (unadjusted and adjusted by SG) was tested for normality using the Shapiro-wilk test. A non-parametric approach was followed due to evidence of skewness (Figure S1). The Wilcoxon rank sum test was used to compare fluoride concentrations in tap water based on tap water use (%) and water filtration. Spearman’s rank correlation was employed to assess the strength of the relationships between fluoride in tap water and fluoride in toenail and urine, while stratifying by residential tap water use (< 25%, 25–50% vs. 50–75% and 75–100%) and water filtration. We also included a stratification by residential tap water use considering < 25%, vs. 25–50%, 50–75% and 75–100%. To quantify the overall contribution of tap water to biomarker levels, unadjusted log-log linear regression models using natural log-transformed variables were used to calculate the geometric mean ratio (GMR) of each biomarker fluoride concentration per doubling of tap water fluoride. The linearity assumption of the log-log regression models was verified using standard residuals versus fitted plots. Additionally, a categorical model was used to calculate GMRs comparing biomarker concentrations between participants with tap water fluoride ≥ 0.7 mg/L versus < 0.7 mg/L. In sensitivity analyses, we re-assessed the strength of the relationships between fluoride in tap water and fluoride in toenail and urine by excluding participants that reported consumption of tea with caffeine (including green tea) during pregnancy. To evaluate changes in toenail fluoride content over time, we restricted the sample to participants with both prenatal and postpartum toenail samples (*n* = 171), then differences in toenail fluoride content between the two time points were compared using a paired Wilcoxon signed-rank test. All analyses were conducted in R version 4.4.2 [39].

## Results

### Study Sample Characteristics

Participants with toenail fluoride data (*n* = 389) and those with urinary fluoride data (*n* = 284) are described in Table 1. The median age at enrollment was 31 years in both groups. Most participants had completed a college or postgraduate degree. For parity, participants were almost evenly distributed between having no prior births or one prior birth. Rates of smoking during pregnancy were low (~ 5.0%). Median body mass index (BMI) values were similar between groups. Most participants indicated residential tap water use “50–75%, 75–100%”. One-third reported using a water filter. Regarding tea consumption, the proportions were similar across both groups, with 47% of the toenail sample and 44% of the urine sample reporting consumption during pregnancy.

**Table 1:** Sample characteristics

Characteristic	Participants with toenail fluoride *N* = 389	Participants with urinary fluoride *N* = 284
Age at study enrollment (years), Median (IQR)	31 (6.3)	31 (5.4)
Education, number (%)		
At least some college	115 (30%)	69 (24%)
College or postgraduate	264 (68%)	207 (73%)
Unknown	10 (2.6%)	8 (2.8%)
Pre-pregnancy BMI, Median (IQR)	24.8 (6.7)	24.1 (6.5)
Parity, number (%)		
0 births	159 (41%)	127 (45%)
1 birth	154 (40%)	104 (37%)
≥ 2 births	74 (19%)	50 (18%)
Unknown	2 (0.5%)	3 (1.1%)
Smoked during pregnancy, number (%)		
Yes	20 (5.1%)	14 (4.9%)
No	362 (93%)	260 (92%)
Unknown	7 (1.8%)	10 (3.5%)
Residential tap water use, number (%)		
<25%, 25–50%	83 (21%)	55 (19%)
50–75%, 75–100%	178 (46%)	146 (51%)
Unknown	128 (33%)	83 (29%)
Residential water filtration, number (%)		
Yes	119 (31%)	94 (33%)
No	270 (69%)	190 (67%)
Tea consumption*, number (%)		
Yes	183 (47%)	126 (44%)
No	177 (46%)	137 (48%)
Unknown	29 (7.5%)	21 (7.4%)

*Based on the food frequency questionnaire’ item: “*Tea with caffeine (8 oz. cup)*,* including green tea*”. Abbreviations: *BMI *= body mass index, *IQR *= interquartile range

### Fluoride in Toenail, Urine and Residential Tap Water

Summary statistics for fluoride concentrations in toenail, urine, and residential tap water are presented in Table 2. Toenail fluoride content had a median (IQR) of 2.60 (1.71) µg/g, with values ranging from 0.54 to 31.90 µg/g. Median (IQR) urinary fluoride concentrations were 0.34 (0.45) mg/L unadjusted and 0.39 (0.38) mg/L after adjustment for specific gravity. Residential tap water fluoride concentrations were similar among participants with either biomarker, with overall median (IQR) values of 0.19 (0.38) mg/L and wide ranges observed across both subgroups. The proportion of participants with fluoride concentrations exceeding 0.7 mg/L in their tap water was 19% (73 of 389) and 16% (46 of 284 samples) for the groups with toenail and urine samples, respectively. The proportion exceeding the 0.7 mg/L threshold for all water samples was 18% (80 of 436 samples).

**Table 2:** Summary statistics for fluoride biomarkers and residential tap water fluoride

Characteristic	*N*	Min	p25	p50	p75	Max
Toenail fluoride (µg/g)	389	0.54	1.84	2.60	3.55	31.90
Urinary fluoride (unadjusted) (mg/L)	284	0.03	0.17	0.34	0.62	4.82
Urinary fluoride (adjusted by specific gravity) (mg/L)	284	0.02	0.23	0.39	0.62	3.92
Tap water fluoride concentration (mg/L)						
in toenail sample	389	0.04	0.10	0.19	0.49	8.23
in urine sample	284	0.02	0.10	0.19	0.46	5.34

*N* = Number of observations, *Min *= Minimum, *p25 *= 25th percentile, *p50 *= 50th percentile (median), *p75 *= 75th percentile, *Max *= Maximum

### Comparison of Fluoride Biomarkers and Fluoride in Tap Water by Percent of Residential Tap Water use and Filtration

For participants with both filtered and unfiltered water samples (*n* = 47), no significant differences were observed in the fluoride concentration in filtered versus unfiltered tap water as shown in Table S1. When comparing fluoride in toenail, urine, and water by the percent of residential tap water use or residential filtration, there were no significant differences (Table 3). The distributions of fluoride in tap water were similar among those who did and did not use water filtration in their homes.

**Table 3:** Comparison of fluoride biomarkers and fluoride in tap water by percent of residential tap water use and filtration

	Residential tap water use					Residential filtration				
	< 25%, 25–50%		50–75%, 75–100%			Yes		No		
	n	Median (IQR)	n	Median (IQR)	p-value	n	Median (IQR)	n	Median (IQR)	*p*-value
Toenail fluoride (µg/g)	83	2.35 (1.21)	178	2.50 (1.59)	0.34	119	2.76 (1.99)	270	2.43 (1.65)	0.09
Urinary fluoride (adjusted by specific gravity) (mg/L)	55	0.43 (0.32)	146	0.39 (0.38)	0.15	94	0.35 (0.30)	190	0.40 (0.40)	0.06
Tap water fluoride concentration (mg/L)										
in toenail sample	83	0.16 (0.28)	178	0.17 (0.38)	0.91	119	0.17 (0.30)	270	0.19 (0.48)	0.19
in urine sample	55	0.16 (0.29)	146	0.19 (0.37)	0.59	94	0.19 (0.32)	190	0.19 (0.38)	0.87

*n*=number, *IQR *= interquartile range. *p*-values were calculated using the Wilcoxon rank sum test

### Relationships between Fluoride in Residential Tap Water, Toenail, and Urine Samples

**Fig. 2:**
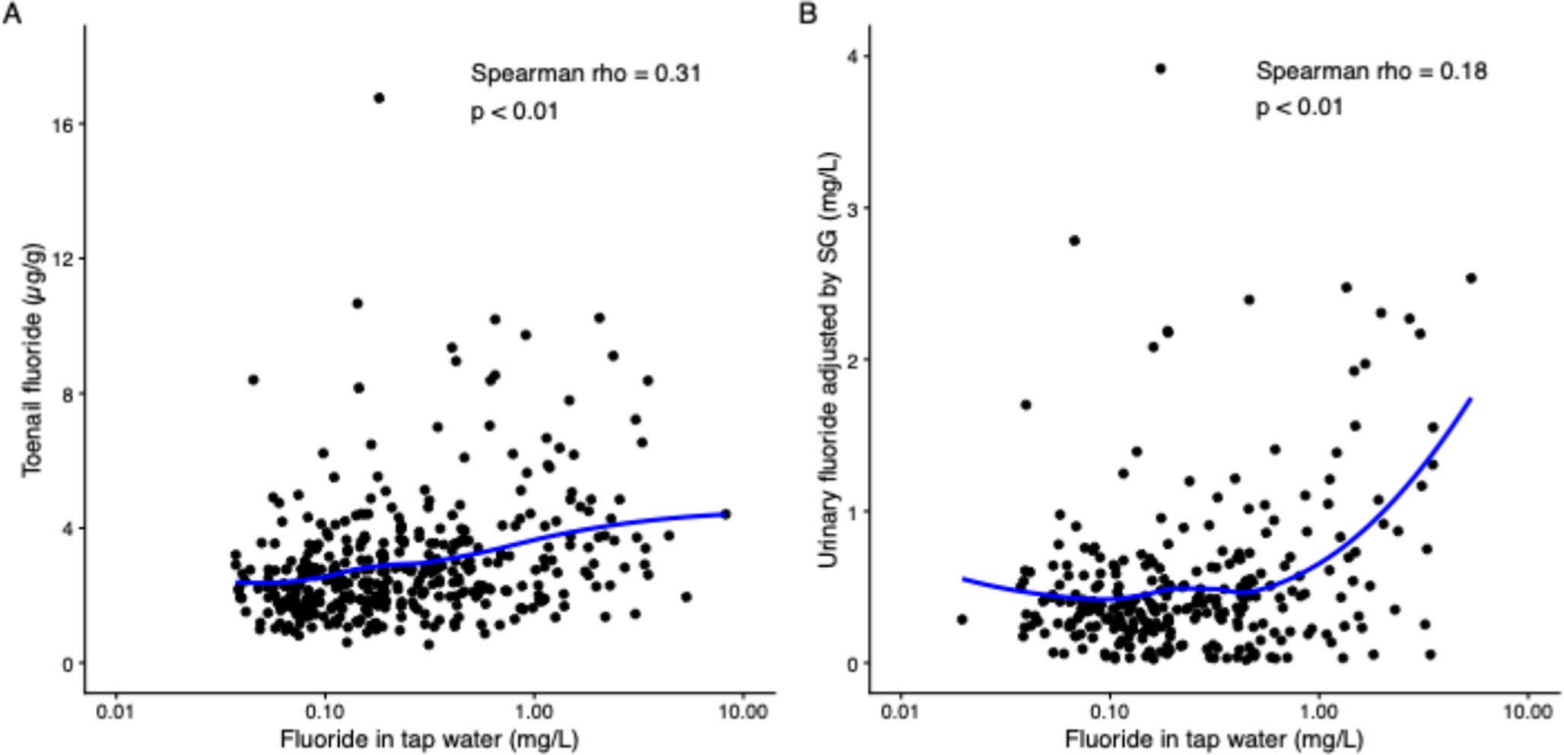
(**A**) Fluoride in residential tap water (mg/L) and toenail fluoride content (µg/g). (**B**) Fluoride in residential tap water (mg/L) and urinary fluoride concentration (mg/L). Note: One observation with toenail fluoride content = 31.90 µg/g was excluded from panel A for visualization. The x-axis is presented on a base-10 logarithmic scale. Flexible loess smoothed lines are highlighted in blue

A moderately strong positive correlation was found between fluoride in residential tap water and fluoride in toenails (Spearman rho: 0.31; *p* < 0.01; Fig. 2A). A weaker, but statistically significant correlation was observed between fluoride in residential tap water and urinary fluoride adjusted by specific gravity (Spearman rho: 0.18; *p* < 0.01; Fig. 2B).

In overall log-log linear regression models, a doubling of tap water fluoride was associated with a 10.3% increase in the geometric mean of toenail fluoride (GMR = 1.10; 95% CI: 1.07, 1.14; *p* < 0.01) and a 10.2% increase in specific gravity-adjusted urinary fluoride (GMR = 1.10; 95% CI: 1.03, 1.18; *p* < 0.01). Based on these regression models, tap water fluoride explained 9.3% of the variance in toenail fluoride and 2.5% of the variance in specific gravity-adjusted urinary fluoride. Participants with tap water fluoride ≥ 0.7 mg/L exhibited significantly higher geometric mean concentrations of toenail fluoride (GMR = 1.37; 95% CI: 1.20, 1.56; *p* < 0.01) and specific gravity-adjusted urinary fluoride (GMR = 1.74; 95% CI: 1.29, 2.36; *p* < 0.01) compared to those below 0.7 mg/L.

Table 4 presents Spearman correlation coefficients for fluoride concentrations in residential tap water and each biomarker, stratified by reported tap water use and residential filtration. Among participants who reported using tap water “50–75%, 75–100%”, statistically significant positive correlations with water fluoride were observed for urinary fluoride adjusted by specific gravity (rho = 0.21; *p* = 0.01) and toenail fluoride (rho = 0.31: *p* < 0.01). Correlations were relatively weaker among participants who reported using tap water “<25%, 25–50%”, particularly for urinary fluoride. These findings were similar when a cut-off of “<25%”, vs “25–50%, 50–75% and 75–100%” was used (Table S2). Regarding residential filtration, correlations were stronger among those who did not report having a water filter compared to those who did, for toenail fluoride (rho = 0.34 vs. 0.26). Residential filtration had a negligible impact on the strength of the correlation between water fluoride and urinary fluoride (rho = 0.18 vs. 0.16).

**Table 4:** Correlation analysis between residential tap water fluoride concentration and fluoride biomarkers, stratified by tap water use and filtration

	Residential tap water use						Residential filtration					
	< 25%, 25–50%			50–75%, 75–100%			Yes			No		
	n	rho	p-value	n	rho	p-value	n	rho	p-value	n	rho	p-value
Toenail fluoride (µg/g)	83	0.27	0.01	178	0.31	< 0.01	119	0.26	< 0.01	270	0.34	< 0.01
Urinary fluoride (adjusted by specific gravity) (mg/L)	55	0.06	0.64	146	0.21	0.01	94	0.16	0.12	190	0.18	0.01

*rho from Spearman’s rank correlation coefficient. Abbreviations: n= number

### Sensitivity Analyses

When excluding participants who reported tea consumption, the correlation between fluoride in tap water and toenails became slightly weaker (Spearman rho: 0.25; *p* < 0.01). On the contrary, the correlation between fluoride in tap water and urinary fluoride adjusted by specific gravity became stronger (Spearman rho: 0.34; *p* < 0.01). The correlation between water fluoride and urinary fluoride showed a pronounced increased for those reporting tap water use “50–75%, 75–100%” during pregnancy (Spearman rho: 0.51; *p* < 0.01; Table S3).

In a sensitivity analysis restricted to participants with both toenail samples available (*n* = 171), the median (IQR) toenail fluoride content was significantly higher during the prenatal period [2.85 (2.15) µg/g] compared to the postpartum period [1.99 (1.16) µg/g] (*p* < 0.01; Figure S2).

## Discussion

The primary objective of this study was to assess the strength of the relationships between fluoride concentration in tap water and biomarkers of fluoride exposure (using toenail and urine fluoride concentrations) in a cohort of pregnant participants in the USA. While the median fluoride in tap water (0.19 mg/L) was well below the U.S. Health and Human Services recommendation of 0.7 mg/L, 18% of participants had water fluoride concentrations higher than that threshold. Our findings showed significant positive correlations between fluoride in tap water and fluoride in toenails and urine. These correlations were stronger for toenails than urine (rho: 0.31 vs. 0.18). Furthermore, when quantifying the magnitude of these relationships, a doubling of tap water fluoride concentration was associated with an approximate 10% increase in the geometric mean of both toenail and urinary fluoride. Consistent with this, participants with tap water fluoride exceeding 0.7 mg/L exhibited significantly higher biomarker concentrations. Together, these findings support toenail fluoride as a reliable biomarker of fluoride exposure during pregnancy.

This study included NHBCS participants, recruited from prenatal clinics in New Hampshire, USA. A unique aspect of this study sample is that their primary household water source consisted of unregulated private water systems. This is relevant when comparing the results of the present study with previous studies on fluoride exposure during pregnancy in the USA, such as those conducted in Northen California [18], and in five states of the Midwest USA: Indiana, Kansas, Kentucky, Missouri and Ohio [19] where participants lived primarily in areas with regulated, public water systems. One key methodological difference is that these prior studies used public water data to retrieve the fluoride concentrations, mainly based on participants’ home address, while NHBCS employed individual household water measures, thereby, providing a more accurate estimate of exposure. The study in Northen California reported a range of community water fluoride concentrations of 0.02 to 1.00 mg/L and the study in the Midwest observed a median of 0.9 mg/L (IQR: 0.8 to 0.9). In contrast, the median fluoride concentration in tap water in this study was lower (0.19 mg/L), but the range was substantially wider (0.02 to 8.23 mg/L). This difference may be explained by the variability in naturally occurring water fluoride on unregulated private water systems used by the participants [26].

To our knowledge, this is among the first studies reporting the content of fluoride in toenails from pregnant participants in the USA. On the other hand, urinary fluoride has been widely used as a biomarker for fluoride exposure during pregnancy, mainly using spot or single void collection [8]. Although urinary fluoride collected as a single void has been accepted as a reliable population/community measure to assess recent fluoride exposure [40], toenail fluoride has the advantage of providing a measurement of longer-term exposure, which has increased its use in epidemiological research [25]. However, toenails as biological samples can present some limitations, such as external contamination [21] and reduced reliability when insufficient sample mass is used for fluoride analysis [32], which may limit the comparability across studies. Fluoride is incorporated into the nail’s matrix from the circulation, and its content in nails depends on factors that influence the nail’s growth rate and length [22]. Although previous studies have reported no significant changes on nail growth or thickness during pregnancy [41], our sensitivity analysis revealed significantly higher toenail fluoride content during the prenatal period compared to the postpartum period. Future studies specifically assessing the influence of the changes of fluoride metabolism during pregnancy on toenail fluoride are needed to further assess its reliability as an exposure biomarker in this population.

Prior studies from the USA [42] and Portugal [43] measured fluoride in toenails in adult non-pregnant female participants. The study in the USA included 482 participants from the Nurses’ Health Study and examined toenail fluoride in relation to hip and forearm fractures in women. They reported mean fluoride content in toenail from controls of 4.5 µg/g [42]. In a study that included 63 women in Portugal, toenail fluoride content ranged from 0.67 µg/g in areas where drinking water contained 0.29 mg/L of fluoride to a mean of 2.75 µg/g in areas with 1.71 mg/L of fluoride in drinking water [43]. Of relevance to the current study, they reported a Spearman correlation of 0.50 between toenail fluoride and daily fluoride dietary intake. In comparison, the present study found a weaker correlation of 0.31 with water fluoride concentration alone, without accounting for estimated water consumption or other dietary sources. These findings underscore that biomarkers reflect total fluoride intake from all sources; thus, correlations with water fluoride may be attenuated when non-water sources of fluoride exposure represent an appreciable fraction of exposure (e.g., consumption of high-fluoride foods and beverages).

There are several studies that have reported maternal urinary fluoride in various populations including in the USA [18, 19, 44], Mexico [20, 45, 46], Canada [17], Denmark [47, 48], Spain [49], Sweden [50], Poland [51], India [52, 53] and Bangladesh [54]. Notably, the studies from Denmark [47] and Sweden [50] also included populations exposed to naturally occurring fluoride in water, reporting median maternal urinary fluoride of 0.52 mg/L and 0.71 mg/L, respectively. In the USA, studies have reported median maternal urinary fluoride concentrations (adjusted by specific gravity) of 0.65 and 0.80 mg/L during the first and third trimesters, respectively, among participants in California [18], and a median of 1.0 mg/L among participants from five Midwestern states [19]. For comparison, the median maternal urinary fluoride observed in this study (0.39 mg/L) was lower than in those prior cohorts. This difference is likely attributable to our sample’s low water fluoride concentrations; at least 75% of participants had tap water levels ≤ 0.46 mg/L, which is considerably lower than the levels reported in previous studies [19]. The implication of this finding is that non-water dietary sources may exert a greater influence on urinary fluoride variability. This was supported by our regression models, which indicate that water fluoride explains only 2.5% of the variance in urinary fluoride. Notably, the study in the Midwest USA reported Spearman’s rank correlation 0.29 with self-reported water consumption [19], which is slightly higher than that found in the present study, 0.21 when considering tap water consumption “50–75%, 75–100%” during pregnancy. This relatively weak correlation observed in our cohort suggests that water fluoride is only a modest predictor of urinary fluoride. This finding reinforces the likelihood that other fluoride sources contribute substantially to total exposure – an important consideration when interpreting biomarkers that reflect short-term exposure to fluoride, especially when only spot urine sample is used.

During pregnancy, tea consumption has been previously reported to be significantly correlated to maternal urinary fluoride levels [17]. Krishnankutty et al. [48] highlighted the risk of increased fluoride exposure due to tea consumption during pregnancy and reported significantly higher concentrations of urinary fluoride in those reporting higher tea consumption. In our sensitivity analyses, we observed that the correlation between fluoride in tap water and maternal urinary fluoride for those using tap water “50–75%, 75–100%” became notably stronger (0.21 vs. 0.51) when we excluded participants reporting tea consumption. This observed change in the strength of the correlation coefficient suggests that the consumption of tea may have influenced urinary fluoride levels, and this could attenuate the correlation with water fluoride levels. In a Canadian study, participants who reported that they drank green, black, or white tea within 24 h of providing a urine sample had 51% higher mean urinary fluoride levels than those who did not (1.31 vs. 0.87 mg/L) [55]. Interestingly, when excluding tea drinkers, the correlation for toenail fluoride seemed less impacted, which reinforces the use of toenail as a longer-term biomarker being potentially less susceptible to variations in fluoride intake in the hours preceding specimen collection.

The present study has some limitations. First, we did not account for toenail growth rate, length or from which toe excluding the big toes, which may influence fluoride detection in toenails [22]. Second, a spot urine sample was collected during the second trimester. Previous studies have reported variation in fluoride concentrations across multiple urine samples collected over the course of pregnancy [17, 20, 45, 46], which may reflect changes in fluoride metabolism during pregnancy [6]. Third, participants’ residential water fluoride levels may not reflect the fluoride levels in other places where they consume water, which could introduce measurement error into our analyses. Furthermore, we did not consider the frequency and volume of tap water consumed to estimate an individual’s dose from water fluoride concentrations as this is challenging to assess over longer versus short time periods. Finally, while our sensitivity analyses accounted for tea consumption, we did not include other dietary sources of fluoride, such as seafood, which can contribute to overall dietary intake in some populations [28, 29]. The non-inclusion of other fluoride dietary sources may increase the risk for residual confusion. However, a separate comprehensive dietary analysis of this cohort indicates that seafood consumption was found to be very low among these participants (Tamayo-Cabeza et al., submitted), which limits its potential impact as a major confounder in this analysis.

## Conclusions

In a rural New England population living in residences served by private, unregulated water systems, we found water fluoride concentrations positively correlated with both urine and toenail fluoride. Correlations were relatively strongest between toenail fluoride and tap water fluoride, likely because toenails reflect longer-term fluoride exposure compared with urine, making them less sensitive to variations introduced by consuming high-fluoride foods or beverages before sample collection. Fluoride concentrations in toenails show potential as a reliable biomarker of fluoride exposure during pregnancy. Future research should aim to validate these findings in diverse populations of pregnant women, particularly those residing in areas with unregulated water systems and varying levels of fluoride exposure.

## Supplementary Information

Below is the link to the electronic supplementary material.

Supplementary Material 1 (DOCX 67.9 KB)

## Data Availability

The data that support the findings of this study may be available from MRK, upon request.
