# Supplementary material for: Relationships between Toenail, Urinary, and Drinking-water Fluoride Concentrations in a Pregnancy Cohort using Private Water Systems in the United States
Source: Biol Trace Elem Res. 2026 Jun 17;204(9):7201–11. doi: 10.1007/s12011-026-05182-7 (PMC13437757; doi:10.1007/s12011-026-05182-7)
Supplement: Supplementary file 1 [file 12011_2026_5182_MOESM1_ESM.docx]

**Figure S1** **A.** Fluoride concentration in residential tap water (mg/L). **B.** Toenail fluoride content (µg/g). **C.** Urinary fluoride concentration (mg/L). **D.** Urinary fluoride concentration adjusted by specific gravity (mg/L).

**Figure S2.** Comparison of toenail fluoride content (µg/g) between prenatal and postpartum collections among participants with both samples available (n=171). Toenail fluoride content was significantly higher during pregnancy (paired Wilcoxon test, p < 0.01).

**Table S1** Comparison of fluoride concentration in residential tap water for participants with both unfiltered and filtered water samples

|  | Unfiltered sample | | Filtered sample | | |
| --- | --- | --- | --- | --- | --- |
|  | n | Median (IQR) | n | Median (IQR) | p-value |
| Fluoride in tap water (mg/L) | 47 | 0.20 (0.31) | 47 | 0.15 (0.28) | 0.14 |

Abbreviations: n= number, IQR = interquartile range.

**Table S2** Correlation analysis between tap water fluoride concentration and fluoride biomarkers, stratified by tap water use with <25% vs ≥25% cut-off

|  | Residential tap water use | | | | | |
| --- | --- | --- | --- | --- | --- | --- |
|  | <25% | | | 25-50%, 50-75%, 75-100% | | |
|  | n | rho | p-value | n | rho | p-value |
| Toenail fluoride (µg/g) | 35 | 0.26 | 0.13 | 226 | 0.30 | <0.01 |
| Urinary fluoride (adjusted by specific gravity) (mg/L) | 23 | 0.11 | 0.61 | 178 | 0.20 | 0.01 |

* Note: rho from Spearman’s rank correlation coefficient. Abbreviations: n= number.

**Table S3** Correlation analysis between tap water fluoride concentration and fluoride biomarkers, stratified by tap water use and filtration, excluding participants reporting tea consumption

|  | Residential tap water use | | | | | | Residential filtration | | | | | | | |
| --- | --- | --- | --- | --- | --- | --- | --- | --- | --- | --- | --- | --- | --- | --- |
|  | <25%, 25-50% | | | 50-75%, 75-100% | | | Yes | | | No | | | |  |
|  | n | rho | p-value | n | rho | p-value | n | rho | p-value | n | rho | p-value |  |  |
| Toenail fluoride (µg/g) | 40 | 0.16 | 0.32 | 79 | 0.27 | 0.02 | 55 | 0.20 | 0.13 | 122 | 0.30 | <0.01 |  |  |
| Urinary fluoride (adjusted by specific gravity) (mg/L) | 28 | 0.14 | 0.48 | 70 | 0.51 | <0.01 | 43 | 0.33 | 0.03 | 94 | 0.34 | <0.01 |  |  |

* Note: rho from Spearman’s rank correlation coefficient. Abbreviations: n= number.
